# Nanofluids and Nanofluidics

**DOI:** 10.3390/nano12172914

**Published:** 2022-08-24

**Authors:** S. M. Sohel Murshed

**Affiliations:** Department of Mechanical Engineering, Instituto Superior Tecnico, University of Lisbon, 1049-001 Lisbon, Portugal; smurshed@tecnico.ulisboa.pt

Both nanofluids and nanofluidics are popular research fields that have attracted tremendous research interest in recent years. This is mainly due to their potential applications in many important fields ranging from energy, electronics and MEMS to biomedical fields. The current trend in technological developments towards devices being smaller and faster raises a lot of technical challenges, particularly those related to thermal management. For instance, small and high-performance devices also generate very high heat flux, and conventional coolants and cooling techniques are increasingly falling short of meeting these high and fast cooling needs. Here, nanofluids and nanofluidics can play a major role in overcoming such challenges (e.g., cooling) related to small high-tech devices and systems. Therefore, the launch of this Special Issue of *Nanomaterials*, covering research and development within these popular and important fields, is timely. A total of nine high-quality articles, including a review article [1] and other articles [2,3,4,5,6,7,8,9] covering wide ranges of research topics from renowned groups across the globe, were published in this Special Issue.

The first article is a review article [1] that provides the state of the art in experimental research on the natural convection heat transfer performances of nanofluids (including hybrid nanofluids) in different geometries from thermal management and conversion perspectives. In addition to briefly reviewing and discussing nanofluids’ preparation, stability evaluation and thermophysical properties, both active and passive techniques employed to enhance the thermal convection performance of nanofluids in different geometries are highlighted and discussed in this extensive review [1].

As the stability of nanofluids is a key challenge for nanofluids’ real-world applications, Primo et al. [2] reported an experimental study assessing the stability of several dielectric nanofluids under realistic transformer operating conditions. They demonstrated that the stability of these dielectric nanofluids strongly depends on several factors, including the working temperature, the types of nanoparticles as well as on the manufacturing procedure.

Another very important factor in nanofluids’ application is their rheological properties, and Vallejo et al. [3] evaluated the influence of six carbon-based nanomaterials on the rheological properties of ethylene glycol/water mixture (50:50 vol.%)-based nanofluids. Shear-thinning behavior was reported, and their dependencies on carbon-based nanomaterial, concentration and temperature were also analyzed in this article [3].

Although nanofluids have shown great potential in heat exchange systems, the presence of nanoparticles in a fluid can affect the formation and attachment of inorganic scales on the surfaces of heat exchangers, hindering their performance. Thus, the effect of carbon nanoparticles on the crystallization of calcium carbonate in aqueous solution was experimentally investigated by Wan et al. [4], who found that nanoparticles (carbon) changed the crystal form of the precipitated calcium carbonate, resulting an excellent surface scale inhibition performance for heat exchange surfaces.

In another study, Chereches and Minea [5] reported the measured electrical conductivity of conventional and hybrid nanofluids containing three different oxide nanoparticles (Al_2_O_3_, TiO_2_ and SiO_2_) in water. The effects of nanoparticle concentration and fluid temperatures were experimentally studied in addition to introducing empirical correlations for the prediction of the electrical conductivity of those nanofluids.

Shah and co-workers [6] examined the stability, thermal conductivity and rheological properties of 2-D tungsten disulphide nanoparticles dispersed in ethylene glycol. The authors found a reduction in the dynamic viscosity of up to 10.5% due to an increase in temperature (from room temperature to up to 70 °C) and an enhancement in the thermal conductivity by up to 6.9%, indicating their advanced heat transfer applications.

As nanofluids have great potential for use in hydrodynamic journal bearings used in heavy industry machinery, a numerical study on the performance of nanofluids as lubricant in high-load journal bearings was reported by Sadabadi and Nezhad [7]. Their results demonstrated an improvement in load-carrying capacity through the use of tungsten disulfide (WS_2_)/oil nanofluid, and they confirmed the potential of this nanofluid for use in journal bearings in heavy industry machinery.

Nanofluids exhibit enhanced boiling heat transfer performance, meaning they have great prospects in many phase-change-based thermal management applications. In this regard, an experimental and numerical study regarding the pool boiling of nanofluids on biphilic surfaces was reported by Freitas et al. [8], and an evident benefit of using biphilic patterns with well-established distances between the superhydrophobic regions was demonstrated.

Another interesting study was presented by Taha-Tijerina and co-workers [9], who applied nanotori structures in various filler fractions as reinforcements to evaluate the behavior of water-based and oil-based lubricants in thermal transport. They reported a highly effective and favorable improvement in the heat transport of both lubricants with nanotori structures.

The total accumulated number of citations (to date) of the articles published in this Special Issue is already 111, which indicates the high quality and impact of these articles. I also strongly believe that the articles within this Special Issue will continue to be useful for researchers working in these fields.
